# Development of an interdisciplinary training program about chronic pain management with a cognitive behavioural approach for healthcare professionals: part of a hybrid effectiveness-implementation study

**DOI:** 10.1186/s12909-024-05308-2

**Published:** 2024-03-22

**Authors:** Wouter Munneke, Christophe Demoulin, Jo Nijs, Carine Morin, Emy Kool, Anne Berquin, Mira Meeus, Margot De Kooning

**Affiliations:** 1 Department of Physiotherapy, Human Physiology and Anatomy, Faculty of Physical Education and Physiotherapy, Vrije Universiteit Brussel, Brussels, Belgium; 2 Pain in Motion International Research Group (PiM), https://paininmotion.be/; 3 Department of Sport and Rehabilitation Sciences, University of Liège, Liege, Belgium; 4 Department of Health and Rehabilitation, Unit of Physiotherapy, Institute of Neuroscience and Physiology, University of Gothenburg, Gothenburg, Sweden; 5 Department of rehabilitation medicine and physiotherapy, University Hospital Brussels, Brussels, Belgium; 6 Société Scientifique de Médecine Générale (SSMG), Brussels, Belgium; 7 Domus Medica, Antwerp, Belgium; 8 Department of Physical and Rehabilitation Medicine, Cliniques universitaires Saint-Luc, Brussels, Belgium; 9 MOVANT research group, Department of Rehabilitation Sciences and Physiotherapy, Faculty of Health Sciences and Medicine, University of Antwerp, Antwerp, Belgium

**Keywords:** Persistent pain, Education, Implementation, Health personnel, Communication, Health planning, Expert Opinion, Behavior and behavior mechanisms

## Abstract

**Background:**

Many applied postgraduate pain training programs are monodisciplinary, whereas interdisciplinary training programs potentially improve interdisciplinary collaboration, which is favourable for managing patients with chronic pain. However, limited research exists on the development and impact of interdisciplinary training programs, particularly in the context of chronic pain.

**Methods:**

This study aimed to describe the development and implementation of an interdisciplinary training program regarding the management of patients with chronic pain, which is part of a type 1 hybrid effectiveness-implementation study. The targeted groups included medical doctors, nurses, psychologists, physiotherapists, occupational therapists, dentists and pharmacists. An interdisciplinary expert panel was organised to provide its perception of the importance of formulated competencies for integrating biopsychosocial pain management with a cognitive behavioural approach into clinical practice. They were also asked to provide their perception of the extent to which healthcare professionals already possess the competencies in their clinical practice. Additionally, the expert panel was asked to formulate the barriers and needs relating to training content and the implementation of biopsychosocial chronic pain management with a cognitive behavioural approach in clinical practice, which was complemented with a literature search. This was used to develop and adapt the training program to the barriers and needs of stakeholders.

**Results:**

The interdisciplinary expert panel considered the competencies as very important. Additionally, they perceived a relatively low level of healthcare professionals’ possession of the competencies in their clinical practice. A wide variety of barriers and needs for stakeholders were formulated and organized within the Theoretical Domain Framework linked to the COM-B domains; ‘capability’, ‘opportunity’, and ‘motivation’. The developed interdisciplinary training program, including two workshops of seven hours each and two e-learning modules, aimed to improve HCP’s competencies for integrating biopsychosocial chronic pain management with a cognitive behavioural approach into clinical practice.

**Conclusion:**

We designed an interdisciplinary training program, based on formulated barriers regarding the management of patients with chronic pain that can be used as a foundation for developing and enhancing the quality of future training programs.

**Supplementary Information:**

The online version contains supplementary material available at 10.1186/s12909-024-05308-2.

## Introduction

Chronic pain affects approximately 20% of the population worldwide [1]. Chronic pain has a tremendous personal and socioeconomic impact: it causes the highest number of years lived with disability [2] and is the largest cause of work-related disability [3, 4]. The intensity, functional impact and persistence of pain are influenced by biopsychosocial factors [5–9]. Factors such as comorbidities, physical well-being, behaviour, psychosocial well-being and environmental aspects can all influence the pain a person experiences [5–9]. This understanding of chronic pain has shifted management strategies from pure biomedical treatments to multimodal approaches acknowledging the complex biopsychosocial nature of chronic pain.

Nonetheless, integrating biopsychosocial chronic pain management is complex. As a consequence, many applied treatments remain biomedically oriented and defined as low-value care [10], resulting in poorer pain, activity and work-related outcomes [11–13]. In addition, patients often consider their treatment to be inadequate [1, 14–16]. With decades of education, dozens of guidelines and many good intentions to improve care, the gap between science and clinical care remains, which limits the implementation of biopsychosocial chronic pain management in clinical practice. There are multifactorial reasons why clinical guidelines are poorly adhered to by HCPs, e.g. lack of knowledge regarding pain and pain management [17–23], HCPs feel that their skills and confidence are insufficient to change their behaviour, which is sometimes also not applicable in their clinical practice [24–27]. Furthermore, patient ability and preferences also affect HCPs’ guideline adherence [21, 28, 29].

Postgraduate training programs could lower these barriers by improving HCPs’ knowledge, skills and confidence to facilitate behavioural change. Studies indicate that educational interventions resulted in more guideline-adherent’ recommendations regarding activity, bed rest and imaging referral [30] and on actual referral behaviour [31] than solely providing clinical guidelines, although French et al. (2013) found significant differences in guideline-adherent imaging recommendations but not in actual imaging behaviour [30]. In addition to improved guideline adherence, training programs are effective in improving HCPs’ knowledge and skills regarding the management of pain with effect sizes ranging from small to large [32–37]. However, this effect can decline over time [38]. Most educational training programs were applied to monodisciplinary groups of HPCs, while there is a need for interdisciplinary training to facilitate interdisciplinary collaboration within healthcare [20, 39, 40]. In addition, interdisciplinary collaboration in clinical practice is associated with improved psychosocial attitudes and might therefore benefit the mid- and long-term effectiveness of training programs [39, 41, 42]. However, little is known about the impact of interdisciplinary postgraduate pain educational training programs, especially when focusing on chronic pain. Given the established need for interdisciplinary educational training programs to improve interdisciplinary collaboration within healthcare [20, 39, 40], the lack of studies examining the impact of interdisciplinary postgraduate chronic pain training educational programs represents a significant knowledge gap. Such interdisciplinary postgraduate chronic pain training programs are also challenging, as they have to be applicable to all HCPs. Here, we aimed to address the significant knowledge gap by developing an interdisciplinary training program about chronic pain for HCPs.

For the reasons outlined above, within this study, we describe the development of an interdisciplinary training program about chronic pain for HCPs. First, an interdisciplinary expert panel was organised to identify barriers and needs expressed by stakeholders for such an interdisciplinary chronic pain training program. Second, the identified barriers and needs of stakeholders for a chronic pain training program were used for the development of an interdisciplinary training program regarding the management of patients with chronic pain. This study is part of a type 1 hybrid implementation study to evaluate the impact of an interdisciplinary training program about chronic pain on HCPs’ knowledge, attitudes, and to assess the determinants of implementation behaviour.

## Methods

The study was approved by an independent Medical Ethical Committee (EC-2021-327) linked to the University Hospital of Brussels, Brussels, Belgium and was in accordance with the Guideline for Reporting Evidence-based practice Educational interventions and Teaching (GREET) [43], Template for Intervention Description and Replication (TIDieR) checklist [44] and Standards for Reporting Implementation Studies (StaRi) Statement [45].

### Belgian context

Belgium is a European country with 11.7 million inhabitants and is divided into three regions: Flanders – official language Dutch -, Brussels official language Dutch and French - and Wallonia – official language French. Belgium has a federal government (Federal Public Service) that manages substantial parts of public health. Each region has its own governance with powers in fields that are connected with its region. In 2019, 7.9% (€37.2 billion) of the Belgian Gross Domestic Product, is spent on health [46]. In 2022, Belgium had approximately 61.858 medical doctors, 41.535 physiotherapists, 13.255 nurses 210.079 dentists, 22.508 pharmacists, 14.478 occupational therapists and 14.641 clinical psychologists [47]. However, these are registered HCPs and do not represent all practising HCPs. Most of the care is coordinated by primary care doctors, and access to a physiotherapist or occupational therapist requires a referral. Care will require expenses by the patient because it is partly reimbursed by health insurance – which is mandatory for all inhabitants. Approximately 23% of the Belgian population has chronic pain [1]. Among primary care doctor practices, chronic pain patients account for 33 to 49% of the consultation, with 81% reporting pain lasting for more than a year [48]. Moreover, pain serves as the primary motive for consultation in 78% of (sub)acute patients and 54% of chronic pain patients [48].

The study consortium consists of three partners: an international research group, Pain in Motion, administratively embedded at VUB in collaboration with Université de Liège, Ghent University, Antwerp University and Université Catholique de Louvain; and two primary care doctors associations - SSMG and Domus Medica - who represent Dutch and French-speaking primary care doctors in Belgium. The Belgian Federal Public Service of Health, Food Chain Safety and Environment funded this study. Together with affiliated healthcare policy organisations, the Federal Public Service was represented in a guidance committee. This committee supervised the progress of the study and provided feedback based on reports and presentations by the study consortium.

## Pain management competencies

Pain management competencies were used as a basis to determine if they were appropriate to guide the development of the training program, to assess the extent healthcare providers meet this standard and as learning outcomes for the training program. The competencies were based on the book Explain Pain [49] which aims to demystify the process of understanding and managing pain. This was requested within the funding application of the Belgian Federal Public Service of Health, Food Chain Safety and Environment. Subsequently, the consortium worked collaboratively to refine and formulate these competencies until consensus was achieved among the members who applied for the grant (JN, CD, MDK, MM, & AB). The pain management competencies were:

1. Understand acute and chronic pain within a biopsychosocial framework
  a Understand the difference between pain and nociception and acute and chronic pain.
  b Recognize that the purely biomedical model is out-of-date and that the biopsychosocial model of pain should be adopted.
2. Assess patients with (chronic) pain comprehensively
  a Use questionnaires and interviews to identify patients’ biopsychosocial factors which might influence pain experience according to the PSCEBSM model [9] (pain–somatic factors – cognitive factors – emotional factors – behavioural factors – social factors – motivation).
  b Assess the patients’ resources, obstacles to improvement, and their “readiness to change”.
3. Integrate contemporary pain science into clinical reasoning in patients with chronic pain
  a Incorporate patients' biopsychosocial factors when making decisions regarding chronic pain type (e.g. nociceptive, neuropathic and/or nociplastic pain), patients’ evaluation and care request.
  b Design multimodal treatment programs, either mono- or interdisciplinary, according to the patients’ representations, beliefs, expectations and needs, e.g. stress self-management program, graded activity program, graded exposure, education/reassurance, etc.
4. Provide tailored and patient-centred strategies to subacute and chronic pain patients
  a Educational strategies:
    i. Understand that pain science education (PSE) is a continuous process;
    ii. Use communication skills to favour therapeutic alliance;
    iii. Master pain neurophysiology and the biology behind different pain mechanisms to be able to explain pain to patients by means of metaphors and tools.
  b Use a patient-centred approach to define specific goals that are meaningful to the patient.
  c Manage obstacles to improve the patient’s motivation to change.
  d Teach patients pain coping skills aligned with the ideas delivered during PSE.
5. Understand the role of HCPs in an interdisciplinary perspective
  a Understand other healthcare disciplines' roles in successfully managing chronic pain.
  b Communicate adequately with other HCPs about the management of chronic pain.

### Interdisciplinary expert panel

Knowing the priority groups’ setting and the barriers and needs to change is essential to achieve successful implementation [50–54]. We selected priority groups with HCPs working in primary care since these are the first HCPs in contact with patients with chronic pain. We selected primary care doctors, (home)nurses, psychologists, physiotherapists, occupational therapists, dentists and pharmacists. Although we focused on priority groups, the training program was accessible for all HCPs.

An interdisciplinary expert panel was organised included 21 experts: a Dutch and a French-speaking expert for each priority group, two pain centre specialists, two heads of pain centres, a member of a patient association and a member of a Belgian organisation that focuses on guideline implementation.

The interdisciplinary expert panel completed an online questionnaire in which they indicated the importance of the established competencies. Additionally, they were asked to provide their perceptions of the extent to which Belgian HCPs already possess the competencies in their clinical practice. Furthermore, the expert panel was asked to formulate barriers and needs relating to training content and the implementation of biopsychosocial chronic pain management with a cognitive behavioural approach in clinical practice within Belgian healthcare, in line with contemporary pain science. They were asked to provide the barriers and needs at the level of HCPs, patients, organisations and the healthcare system. All answers regarding barriers and needs through the online questionnaire were included. The answers were accompanied by a literature search and discussed during the first meeting to provide a deeper understanding of the barriers, needs and specific context variables relevant to the implementation study. We used a framework to guide and organise the barriers and needs, and to characterise interventions and policies to change behaviour [55]. This framework consists of the Theoretical Domain Framework, containing 14 domains regarding behavioural change, which were mapped into the COM-B model. The COM-B model is a guide to design interventions, and include the domains ‘capability’, ‘opportunity’, and ‘motivation’ [56]. Three online meetings with the expert panel were organised, one to discuss the barriers and needs, one to evaluate the patient materials and one to evaluate the training program prior to implementation. The expert panel received an update about the results of the training program after the completion of the implementation process.

### Chronic pain training program

An original and interactive blended learning training program was developed including two e-learning modules and two face-to-face workshops based on the barriers and needs formulated by the literature search and expert panel. The training program aimed to improve HCP’s competencies for integrating biopsychosocial chronic pain management with a cognitive behavioural approach into clinical practice. Both a Dutch and French version was developed. Each e-learning module last approximately 1 h, and the each workshop 7 h. This amount of training hours is commonly applied and reported to be effective in changing knowledge, attitudes and determinants of implementation behaviour [57, 58].

The e-learning modules provided the theoretical basis to the participants and maximised the time for interactions and skills training during the workshops. The two workshops – in interdisciplinary groups - were designed to focus on skill training and practical implementation of biopsychosocial model and improved communication techniques and PSE for a cognitive behavioural approach in clinical practice, because this is applicable and essential to all HCPs [59–66]. Approximately a month was planned between both workshops so participants can practice in their clinical practice and their experience can be discussed during their second workshop. We used a variety of educational methods, such as interactive lessons, video materials, local opinion leaders [67], demonstrations, illustrations, assignments, skills training, clinical reasoning training, goal settings, role playing, case studies and interdisciplinary discussions, and peer- and teacher feedback to improving the learning process [67–70]. Interdisciplinary collaborative exercises were applied to facilitate uniformity in communication and chronic pain management approach, and improved collaboration in clinical practice. These methods were used to reduce the barriers and accommodate the needs formulated by the expert panel to implement the biopsychosocial model, corresponding to HCPs’ current best-evidence approach in line with modern pain sciences [41, 69]. Both workshops included mandatory phases in combination with optional phases that could be adapted to the expectations and needs of the participants.

After participating in the training program, participants were asked if they were interested in sharing their name, work address(es) and contact details. With this information, an interactive map was developed and shared with all participants to improve their interdisciplinary collaboration. The local trainers aimed to facilitate a sustainable change by acting as a chronic pain resource person for the HCPs in the geographic areas after the implementation study.

### Patient materials

Patient materials were developed to support the integration of the biopsychosocial model and PSE in clinical practice and the quality of PSE for patients with chronic pain. The patient materials included posters, a patient booklet – which was an update from an existing PSE booklet [71] - and videos explaining pain were created by collaborating with the Retrain Pain Foundation by making videos from their PSE slides [72]. A panel of five Dutch-speaking and five French-speaking patients with chronic pain were organised to co-design these materials. These patients were recruited from two chronic pain patient organisations and within the university hospital of Brussels (UZ Brussel). The patient panel discussed patients’ needs, information and messages that were important to patients and provided feedback on the developed materials. The patient materials discusses the impact of pain, why we feel pain, the difference between acute and chronic pain, the role of the nervous system and the brain, an overprotective alarm system and contributing factors, and how to manage chronic pain (e.g. improve understanding about pain, beliefs and expectations, active lifestyle, stress management, social life, sleep, positive and negative effects of medication, self-management and the support from HCPs. The patient materials were evaluated based on the following criteria: ‘clarity’, ‘content’, ‘usefulness’, ‘layout’, ‘understandability’, ‘added value or not’, ‘consistency’ and ‘suggestions for improvements’ by the expert panel and patient panel. All materials were updated based on their feedback to improve quality.

### Trainer recruitment and train-the-trainer workshop

Each training was provided by a pair of teachers: an expert teacher and a local expert. The experts were affiliated with the consortium, graduated as HCPs, had experience with teaching, and were familiar with chronic pain, the biopsychosocial model and PSE. The local experts were HCPs working in the geographic area of training implementation and helped to tailor the training program to the local context, i.e. taking into account the sociocultural diversity of the patient population in the geographic area and the local, formal and informal networks of HCPs. The criteria for local trainer were as follows: fluent in Dutch or French, three days a week of work with patients with chronic pain in the geographic areas of implementation, expertise in chronic pain, a biopsychosocial perspective, ability to participate in the train-the-trainer workshop, and ability to provide at least two workshops.

The train-the-trainer workshops were implemented to secure the quality of the trainers and to ensure that the trainers’ knowledge and attitudes were in line with the training content. It included online one-on-one training sessions and discussions about chronic pain organised by the expert trainer with whom the local trainer forms a training duo. This personal train-the-trainer workshop provided the opportunity to adapt it to the needs of the expert and local trainer. In addition, group meeting(s) with other local trainers were organised for more general discussions to ensure that the core of the training program was the same for all training duos. At the end of the train-the-trainer workshop, all trainers completed the Knowledge And Attitudes of Pain questionnaire to assess their level of knowledge and attitudes toward pain in line with modern pain science [73, 74]. Trainers received a fee of €350 for participating in the train-the-trainer workshop and a fee of €600 for each day of provided workshops for HCPs.

### Recruitment of healthcare professionals

We aimed to train 500 HCPs at minimum within a total of 25 groups with approximately 20 to 25 HCPs — five training groups in each implementation area; Antwerp, Gent (both Flanders), Brussels (Brussels), Namur, and Liege (both Wallonia). We prioritised recruitment of HCPs working in the cities where we implemented the training to facilitate interdisciplinary collaboration during and after the training program. If there were still available spots for a training group a month prior to the training date, the recruitment was expanded to a wider geographical area. Therefore, all HCPs in Belgium were eligible to register for the training program. HCPs were recruited through multiple methods and networks. The consortium collaborated with organisations associated with HCPs in primary care, the Federal Public Service, and organisations connected to the study to recruit HCPs. All organisations shared information and flyers on their website, magazines, social media and/or within their network.

Participants received continuing education credits for participating in the training program to stimulate participation. The cost of the training programs was covered within the funding. Therefore, the training was free for participants, making the training also accessible for HCPs with fewer financial resources. In addition, the training program was implemented at various days of the week - Monday to Saturday - and various periods of the day - morning and afternoon or afternoon and evening - so that it enabled most HCP to participate within their work scheme.

### Data collection and evaluation

HCPs were recruited from August 2021 to May 2022 and October 2022 to June 2023. Workshops were organised from October 2021 to June 2022 and March 2023 to July 2023. Within this study, the results of implementing the training program will be analysed and reported in separate papers. These separate papers will report the short and mid-term changes in HCPs’ knowledge, attitudes and guideline adherence regarding chronic pain and HCP’s confidence regarding low back pain. In addition, we will assess HCP’s barriers and needs of integrating the cognitive behavioural approach. Furthermore, HCPs’ training satisfaction will be evaluated after each workshop and after six months. All HCPs who enrolled in the training program were invited to take part in the studies. Each participant was requested to complete an informed consent form.

## Results

### Interdisciplinary expert panels’ perception towards competencies

**Fig. 1:**
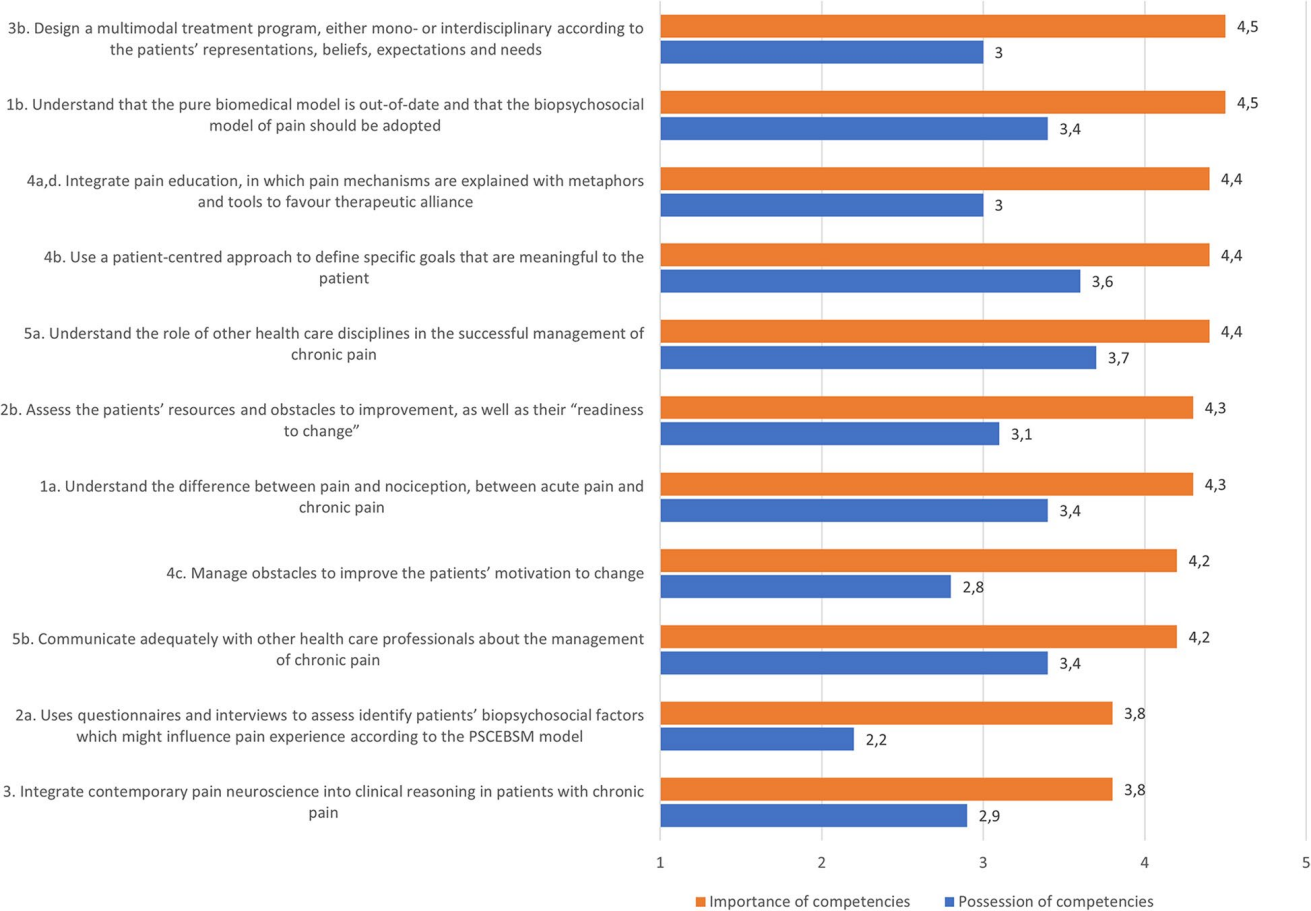
Expert panels’ perception towards the importance and HCPs’ possession of competencies in clinical practice. Importance of competencies: 1 = not important at all, 2 = slightly important, 3 = moderately important, 4 = very important, 5 = extremely important. HCPs’ possession of competencies: 1 = totally disagree, 2 = disagree, 3 = neutral, 4 = agree, 5 = totally agree. Higher scores reflect higher importance and stronger possession of HCPs’ competencies in clinical practice. PSCEBSM = pain – somatic factors - cognitive factors – emotional factors – behavioural factors – social factors – motivation

Within the interdisciplinary expert panel, 17 of the 21 members completed the questionnaire in which they indicated their perceptions of the importance of the competencies and the extent to which Belgian HCPs already possess the competencies in their clinical practice. The expert panel considered 9 competencies as ‘very important’ to ‘extremely important’, see Fig. 1. One of the main competence – ‘integrate contemporary pain neuroscience into clinical reasoning in patients with chronic pain – and a sub competence ‘Use questionnaires and interviews to identify patients’ biopsychosocial factors which might influence pain experience according to the PSCEBSM model - were rated between ‘moderately important’ and ‘very important’. Originally, the questionnaire asked for the importance of integrating contemporary pain neuroscience into clinical reasoning. During the meeting, the expert panel recommended that ‘integrating pain neuroscience into clinical reasoning’ was seen as important when pain science does not solely focus on neurophysiology. Therefore, the competence was changed to ‘pain science’. The importance regarding the use of questionnaires were seen as less important compared to other competencies. Its perception the extent to which Belgian HCPs already possess the competencies in their clinical practice ranged from ‘neutral’ to ‘agree’. This showed that there was large room for improvement on all competencies and that the training program needed to take the low competence in account within the training program. This was done by discussing the importance of the competencies and making it accessible and understandable for HCPs who have less experience and possession of the competencies in their clinical practice.

### Barriers and needs

All 21 members of the interdisciplinary expert panel completed the questionnaire or participated in the meeting relating to stakeholders’ barriers and needs concerning training content and the implementation of chronic pain management with a cognitive behavioural approach in clinical practice within Belgian healthcare, in line with contemporary pain science. The questionnaire and meeting with the interdisciplinary expert panel and literature search identified a large variety of barriers and needs which are presented in the Theoretical Domain Framework for behavioural change linked to COM-B domains, see Table 1.

**Table 1:** Stakeholders’ barriers and needs to implement a chronic pain training program

**COM-B**		**Barriers of HCPs**	**Needs of HPCs**
**Capability**	**Psychological capability (the capacity to engage in the necessary thought processes)**		
	Knowledge	- Lack of knowledge about pain and its characteristics [75–77]^,E^ - Lack of knowledge about an adequate assessment of pain [75] - Lack of knowledge about the biopsychosocial model^E^ - Lack of knowledge about the role, opportunities and barriers of other disciplines^E^ - Not familiar with research and literature [77] - Unfamiliar with adverse effects of narcotics [77] - HCPs have a biomedical perspective [78]^,E^ - Patients have a biomedical perspective, and managing it is difficult [79]^,E^	- Focus training on basic knowledge^E^ - Increase knowledge of pain mechanisms[64] - Increase awareness of all factors to consider when treating a person with chronic pain^E^ - Provide knowledge not only on managing chronic pain but also on preventing patients from developing chronic pain^E^ - Increase knowledge and values of a patient-centred approach^E^ - Increase awareness of social influences (e.g. Friends and family)^E^ - Increase knowledge of the added value of interprofessional learning and working^E^ - Emphasise the importance of a follow-up in treatment programs^E^ - Increase awareness that behaviour change techniques pairing is more effective [65] - Make caregivers aware of the burden of chronic pain^E^ - Provide awareness of insufficient undergraduate education regarding (chronic) pain^E^
	Skills: cognitive and interpersonal	- Difficulty applying psychosocial perspective [76, 78]^,E^ - Difficulty with assessing pain in people with communication difficulties [75] - Problems with interdisciplinary communication [75, 77]^,E^ - Lack of communication and listening skills with patients^E^ - Inability to treat without an established diagnosis [75] - Incompetence to give PSE to patients [75] - Difficulty in dealing with patients with psychological problems [79]^,E^	- Make the training practical^E^ - Integrate effective resources for multimodal pain management^E^ - Practice skills to encourage patients’ self-management^E^ - Practice adapting treatments based on the individual [64] - Practice using metaphors [64] - Train communication strategies/skills [79]^,E^ - Enhance skills to include and assess social and family factors^E^
	Memory, Attention and Decision processes	- HCPs don’t apply a patient-centred approach [76, 77] - No assessment of patient behaviours and beliefs [76] - HCPs apply an inadequate assessment of pain and pain relief [75, 77] - Poor patient reporting in pain management [75] - HCPs work monodisciplinary, no or too few interdisciplinary consultations are made [76]^,E^ - Follow-up of between HCPs are highly variable [76] - Patients have different expectations [76, 79] - Quality of life is not a central objective^E^ - Patients are not open to PSE [79, 80] - Patients have conflicting information^E^ - HCPs have unhelpful attitudes regarding pain [75] - HCPs do not known whether patients ask for pain relief or pain medication [77]	- Use case studies of common problem areas that are applicable to largely the whole group (or can be adapted to the specific caregiver)^E^ - Promote interventions co-facilitated by HCPs with different skills^E^ - Promote interdisciplinary collaboration [81]^,E^ - Take into account therapeutic alternatives^E^ - Practice developing interdisciplinary treatment plans^E^ - Provide the message to take the patient seriously [64] - Encourage acceptance of chronic pain and the biopsychosocial approach^E^ - Provide sufficient time to discuss the participant’s current situation during training^E^ - HCPs think that a therapeutic alliance is important [79]^,E^
	Behavioural regulation	- Too little interest in overly theoretical information^E^ - Patients with fear of pain and consequences communicate less well [75]	- Implement the application and handling of "yellow flags" in the ambulatory setting^E^ - Spend attention and time for interest in meeting other HCPs within the training (build a "social" identity)^E^
	**Physical capability (physical capacity to engage in the activity concerned)**		
	Skills: physical		
**Opportunity**	**Social opportunity (the cultural milieu that dictates the way that we think about things)**		
	Social influence	- Lack of social support for patients^E^ - Lack of society's recognition of the problems of chronic pain^E^ - Cultural/religious differences [77] - Reluctance of patients to report pain [75] - Patients ashamed of symptoms [81] - Experiences and stories of family and friends [75] - Dominance of anaesthesiologists, giving preference to technical treatments [76]	- Create a status for pain management^E^ - Use "Local Opinion Leaders" to increase impact [67] - Let participants discuss chronic pain with colleagues to increase social support [81]
	**Physical opportunity (what the environment facilitates in terms of time, resources, location, physical barriers etc.)**		
	Environmental context and resources	- Lack of adequate training for the issue of 'chronic pain' in the curriculum of training and courses [76, 79]^,E^ - Previous received training was biomedically oriented [80] - Lack of finance/financial compensation (for a comprehensive approach to treating patients with chronic pain) on the Micro, Meso, and Macro level [75–77, 79]^,E^ - Not trained for sociofamilial initiatives [76]^,E^ - Lack of pain specialists and training of teams [76]^,E^ - Excessive workload [76]^,E^ - Inadequate or non-existent education materials [77]^,E^ - No accessibility for patients to receive certain treatments (nonavailability/long travel time) [81] - Uneven geographical distribution of interdisciplinary pain centres [62]^,E^ - Lack of time (Micro, Meso, Macro) [75, 77, 79]^,E^ - Lack of leadership within chronic pain treatment organisations [75, 77] - Available information on chronic pain does not support its implementation, nor does it identify its limits^E^ - Lack of time to start and complete chronic pain training^E^ - Insufficient incentives to support HCPs in such treatments and training initiatives^E^ - (Excessive) cost prevent patients from accessing therapy or cause them to stop treatment early^E^ - Lack of training in dealing with sensitive topics [79]	- Create a network of therapists working in the field of chronic pain; Create peer review groups^E^ - Encourage the use of peer groups for patients with chronic pain^E^ - Develop patient pain educational materials like booklets and videos that are available for patients and HCPs as support for PSE^E^ - Creating postgraduate PSE courses^E^ - Encouraging and creating more available training courses about pain^E^ - More hours of education are needed about pain in training courses^E^ - Make use of apps on smartphones to coach patients and evaluate treatment progress^E^ - Use apps and videos to train caregivers and encourage self-management of caregivers to improve knowledge and skills^E^
**Motivation**	**Reflective motivation (involves self-conscious planning and evaluations)**		
	Social/Professional Role & Identity	- Lack of interest in interprofessional collaboration and to be in a dynamic of integrated care^E^ - Negative attitudes about the role of other disciplines and patients with chronic pain^E^ - Lack of interest in (chronic) pain^E^ - Lack of motivation in patients to participate in long-term treatment pathways^E^ - Lack of awareness of their actions [78, 80] - Different expectations from other HCPs or organisation [78]	- Empower HCPs that their management can include psychological and social factors [79]^,E^ - Use feedback(loop) and action goals to increase the effectiveness of the training program [68]
	Beliefs about capabilities	- Lack of confidence in assessing psychosocial factors and in nonpharmaceutical treatments [78, 79, 81]^,E^ - Less motivated HCPs will be challenging to recruit^E^	- Encourage acceptance that chronic pain management can be ineffective to change pain intensity for some patients and should not be the major goal^E^ - Build confidence for effective therapeutic education^E^
	Optimism		- Provide training with a positive attitude towards pain [81]
	Beliefs about consequences	- Lack of visibility of benefits when collaborating between HCPs in treating patients^E^ - Possible loss of trust in HCPs who have to perform theoretical education^E^ - Anaesthesiologists do not want to go along with guidelines because of increased workload, fear of licensing problems and reduced revenue [77] - Knowledge about the addictive effect of pain medication did not worry HCPs [77]	
	Intentions	- Many HCPs prioritise the importance of other diseases above pain for treatment and training [75] - HCPs seem to lack interest in implementing accumulated knowledge and skills^E^ - Change in behaviour is complex, and resistance from HCPs is expected [80]^,E^ - Lack of willingness and empowerment of HCPs to start and continue a training program^E^ - Not wanting to believe the patient's reported pain [77]	
	Goals	- Lack of goal to encourage patient self-management^E^ - Patients have different values than HCPs ^E^	- Focus your training on improving patients’ quality of life rather than pain management^E^
	**Automatic motivation (involves wants and needs, desires, impulse and reflex responses)**		
	Reinforcement		- Reward HCPs with credits for attending training^E^
	Emotions	- Length of treatment is discouraging for the patient and frustrating for the counsellor [76] - Fear of mistakes when implementing new behaviour [77] - Uncertainty of HCPs^E^ - Patients feel helpless, that they cannot be helped with their problem^E^ - Many HCPs have little trust in the healthcare system^E^	

The overview of barriers and needs relating to the learning processes, competencies and implementation within Belgian healthcare, formulated by the expert panel and literature search

^E^ Formulated by the expert panel, *HCP* Healthcare professional, *PSE* Pain science education

In summary, the barriers and needs reflected the importance of the competencies. Based on the domain of psychological capabilities, the training program needed to improve HCPs’ knowledge and especially skills related to a biopsychosocial approach and interdisciplinary collaboration for the management of patients with chronic pain. It was advised to develop a general chronic pain course which was not too complex, however, there was a stronger need to focus on improving skills than improving knowledge.

The social and physical opportunities domain showed that many environment factors, such as the biomedical perspectives of healthcare and society, and the lack of biopsychosocial education regarding pain, could limit the acceptance of the biopsychosocial model by the participants. In addition, it showed implications for implementation in clinical practice, such as lack of time, resources and support for HCPs and patients. Furthermore, based on the domain of motivation, many HCPs have a lack of interest in the management of patients with chronic pain and interdisciplinary collaboration. In addition, HCPs have less confidence in assessing psychosocial factors, believe that patients have less interest in a biopsychosocial approach and pain education, do not encourage patient goals focused on self-management and quality of life, and have negative emotions relating to pain management.

### Training program

#### E-learning modules

The first e-learning module - of approximately one hour - aimed at achieving competencies 1, 2 and 3 (1. Understand acute and chronic pain within a biopsychosocial framework; 2. Assess patients with (chronic) pain comprehensively; 3. Integrate contemporary pain science into clinical reasoning in patients with chronic pain). It included an “introduction” part explaining the rationale and learning outcomes of the teaching programme and necessary basic theoretical parts, e.g. the impact of chronic pain on patients and society, definitions of pain, physiology of acute pain and chronic pain, the biopsychosocial model, biopsychosocial factors related to chronification and persistence of pain (e.g. stress, anxiety, catastrophising, depression, misbeliefs, insomnia, inactivity, etc.), and types of pain (nociceptive, neuropathic and nociplastic pain).

The second e-learning module aimed at achieving competencies 3, 4 and 5 (3. Integrate contemporary pain science into clinical reasoning in patients with chronic pain; 4. Provide tailored and patient-centred strategies to subacute and chronic pain patients; 5. Understand the role of HCPs in an interdisciplinary perspective).

This module started with a summary of the first e-learning module, after which it introduced patient-centred approach, attitudes, beliefs, motivation and coping of patients, PSE strategies, metaphors, the importance of the words used with patients, goal-setting, obstacles for change, motivational interviewing, self-management and lifestyle, needs and expectations of patients, commonly applied modalities/treatments (e.g. imaging, medication, hands-on techniques, and exercise) and the mono- and interdisciplinary approach in the management of chronic pain.

The e-learning modules used interactive educational methods to activate the participants’ prior knowledge and experience together with an efficient integration with what is new. The content was delivered through video animations, expert interviews and short texts. Reflection questions complemented the content during and after slides and within a test at the end of each session (such as quizzes, multiple-choice tests and open questions on which the participants received automated feedback).

#### Face-to-face workshops

The key aspects of the training program were a biopsychosocial pain assessment, specific patient-centred communication techniques and biopsychosocial treatment programs integrating PSE. The interdisciplinary training program can be found in Online Resource 1.

The first workshop aimed to provide knowledge and skills needed to integrate biopsychosocial (pain) assessment of patients successfully and to give the first introduction to PSE in their practice and to integrate the model and contemporary pain science into clinical reasoning in patients with chronic pain (competencies 1–4). The workshop included lecturing, exercises, interdisciplinary group discussions, and skills training relating to pain assessments, communication, PSE and their barriers and needs to implementing in their clinical practice. After the first workshop, participants received exercises to implement and practice biopsychosocial pain assessment, specific patient-centred communication techniques and PSE in their clinical practice. Participants received a poster providing key messages for patients regarding chronic pain, a patient booklet to support PSE in their clinical setting and the link to the patient videos. All French and Dutch patient materials can be found on the website of Pain in Motion http://www.paininmotion.be/patients/information-about-persistent-pain.

The second workshop aimed to provide the ability to tailor and apply patient-centred strategies to subacute and chronic pain and to understand the role of HCPs from an interdisciplinary perspective. The workshop included lecturing, exercises, interdisciplinary group discussions, and skills training relating to providing PSE, motivational interviewing, patient-centred approach, mono-/interdisciplinary approach and communication between HCPs.

Both workshops contained nine mandatory phases with objectives per phase and two optional phases to adapt the training to the needs of the participants in the group. We evaluated if these phases were applied and achieved through discussions with participants and questions and observations by the trainers. The degree to which the participants were satisfied with the workshops was evaluated by a satisfaction questionnaire after each workshop.

#### Adaptations during the implementation process

The workshops were slightly adapted during the process of implementation. However, the core elements of the workshops remained the same. After the first three workshop groups, a group discussion about the factors influencing pain at the start of the first workshop was removed because participants thought it had less added value in addition to the e-learning modules. Furthermore, participants wanted more time for PSE exercises, so a motivational interviewing exercise was moved to the second workshop. In the second workshop, a motivational interviewing exercise was simplified due to difficulties experienced by participants. Furthermore, during the implementation process, minor adjustments were made in slides to support teachers’ lecturing.

For the first four workshop groups, we aimed to recruit approximately 20 HCPs for each group. However, many participants cancelled last minute due to situations relating to COVID-19. Therefore, in agreement with the trainers, group sizes were increased to approximately 25 for the remaining 11 workshop groups to train a minimum of 300 HCPs but assure the quality of the training program.

## Discussion

The developed interdisciplinary training program regarding the management of patients with chronic pain included a two 7-hour workshops and two e-learning modules - aimed to improve HCP’s competencies for integrating biopsychosocial chronic pain management with a cognitive behavioural approach into clinical practice. A large variety of barriers and needs were formulated - by the interdisciplinary expert panel and literature search - relating to training content and the implementation of chronic pain management with a cognitive behavioural approach in clinical practice. This provided valuable insight into the challenges for the implementation study and for HCPs, which was used to adapt the training program to the Belgian context. This study is part of a type 1 hybrid implementation study to assess the impact of such chronic pain training programs on the knowledge, attitudes and behaviour of HCPs regarding chronic pain management, aiming for higher value care for patients with chronic pain [82].

Recently, Slater et al. (2022) designed a framework in Australia, which is a blueprint for shaping interdisciplinary training about chronic pain with patients, HCPs and pain educators [83]. This framework identified gaps and training targets based on priorities in pain care. Although this study was performed in the Australian context, the identified gaps and training targets are closely aligned with the competencies and content of the training program. It is therefore most likely that our competencies and related barriers and needs are generalizable for many contexts in healthcare worldwide. However, it remains unknown what the optimal dose, intensity and frequency of trainings are needed to address these barriers and needs and to obtain the competencies. Our training program lasted two days, which is a commonly applied duration and has been effective in previous studies to obtain the competencies by improving knowledge, attitudes and behaviour of HCPs [37, 38, 58, 84]. Other studies used training programs ranging from a workshop of multiple hours [32, 84], multiple workshops of a few hours [36] to multiple days [85, 86]. These studies - with both fewer and more hours of workshops - found significant improved knowledge and skills regarding pain knowledge or to educate patients about pain, indicating that obtaining the competencies is feasible. However, the training programs were monodisciplinary and a detailed training program was not published, making it difficult to compare. Konsted et al. (2019) published a brief training program that aimed to support physiotherapists and chiropractors’ integration of the biopsychosocial low back pain management with a cognitive behavioural approach in clinical practice [85]. This training program also included two-day workshops, had similar competences to obtain and a similar mix of theoretical and skills training, was shown to be feasible and effective in changing clinical behaviour [57, 87]. In addition to the training programs reported above, our training program included two e-learning modules to support the workshops, which potentially improved the learning experience and satisfaction of participants [88]. To our knowledge, no other interdisciplinary training program plans are available on the topic of pain.

A strength of this study was the co-design with a large interdisciplinary expert panel who formulated barriers and needs of stakeholders and the use of a framework to organise factors relating to behavioural change [56]. These addressed barriers and needs, together with a blended learning design and interactive teaching methods, improved the quality of the training for HCPs in Belgium [51, 52]. Furthermore, the two-day training program available for all HCPs and targeted for seven disciplines makes it feasible to implement and scale-up for a large population of HCPs and many healthcare systems. Furthermore, the training program was updated during the implementation process to improve the training based on the experiences of the trainers and participants. Another strength is the availability of patient materials - which was developed with a patient panel - as support for HCPs to integrate PSE within clinical practice. Lastly, the training program was implemented in five different areas of Belgium, in two different languages, and is available in Dutch, French and English. However, this study also has several limitations. A more intensive co-design throughout the process with experts and patients may have improved the quality of the training program. Furthermore, the formulated barriers and needs were based on a literature search and the expert panel; no systematic literature review was conducted, which could have resulted in some barriers and needs being missed. Besides, the estimated pre-intervention HCPs’ possession of competencies in their clinical practice was based on the expert panels’ perception and was not based on a large scale survey. Moreover, the training program includes several learning outcomes related to competencies that pose challenges to assess or which are not covered by the initial evaluation plan. Consequently, determining the achievement of some learning outcomes within this implementation study may remain inconclusive.

This study can potentially serve as a foundation for future training, thereby saving the time and resources required to develop training programs de novo. However, training programs need to be further developed and cross-culturally adapted within the geographic areas of implementation. To improve this process, more training programs should be available to facilitate learning from other training programs, e.g. to provide insight into how many hours of practical training is desired or which elements of the training facilitate learning the most effective. By reducing the differences between postgraduate training programs, we might also reduce the differences in knowledge and attitudes between HCPs and potentially improve their interdisciplinary collaboration [89]. Many factors play an important role in the learning experience of HCPs and their behaviour change, and many factors seem poorly understood. Hence, the publication of training programs by projects and studies should be encouraged, and the effectiveness of such training programs and their implementation process in clinical practice should be assessed. Furthermore, studies are needed to compare the effect of interdisciplinary versus monodisciplinary training programs. Although interdisciplinary training groups can facilitate interdisciplinary collaboration, they may introduce variation in the learning effect, as training that focuses on knowledge or skills may not be equally relevant across disciplines [90].

## Conclusion

To address the significant knowledge gap of studies examining the effectiveness of interdisciplinary postgraduate chronic pain training programs, as well as the established need for interdisciplinary training to improve interdisciplinary collaboration within healthcare, an interdisciplinary training program was developed to improve HCP’s competencies for integrating biopsychosocial chronic pain management with a cognitive behavioural approach into clinical practice for the treatment of patients with chronic pain. To do so, an interdisciplinary expert panel was created to identify the barriers and needs of stakeholders for such a chronic pain training program. The identified barriers and needs of stakeholders for a chronic pain management training program were used for the development of the interdisciplinary pain management training program. In addition, the training program can be used as a foundation for developing and enhancing the quality of future training programs.

### Supplementary Information

**Supplementary Material 1.**

## Data Availability

The complete and more detailed training program and materials are available in French and Dutch from the corresponding author on reasonable request.

## Abbreviations

HCP: Healthcare professional
PSE: Pain science education
PSCEBSM-model: Pain – somatic factors - cognitive factors - emotional factors - behavioural factors - social factors – motivation
