# Supplementary material for: Development of an interdisciplinary training program about chronic pain management with a cognitive behavioural approach for healthcare professionals: part of a hybrid effectiveness-implementation study
Source: BMC Med Educ. 2024 Mar 22;24:331. doi: 10.1186/s12909-024-05308-2 (PMC10960450; doi:10.1186/s12909-024-05308-2)
Supplement: Supplementary file 1 — Supplementary Material 1. [file 12909_2024_5308_MOESM1_ESM.docx]

Online Resource: Interdisciplinary training program

**Development of an interdisciplinary training program about chronic pain management with a cognitive behavioural approach for healthcare professionals: part of a hybrid effectiveness-implementation study**

Wouter Munneke^1,2,3^, Christophe Demoulin^3^, Jo Nijs^1,2,4,5^, Carine Morin^6^, Emy Kool^7^, Anne Berquin^8^, Mira Meeus^2,9^, Margot De Kooning^1,2*^

^1^ Department of Physiotherapy, Human Physiology and Anatomy, Faculty of Physical Education and Physiotherapy, Vrije Universiteit Brussel, Brussels, Belgium.

^2^ Pain in Motion International Research Group (PiM), www.paininmotion.be.

^3^ Department of Sport and Rehabilitation Sciences, University of Liège, Liege, Belgium.

^4^ Institute of Neuroscience and Physiology, Department of Health and Rehabilitation, Unit of Physiotherapy, University of Gothenburg, Gothenburg, Sweden.

^5^ Chronic pain rehabilitation, Department of rehabilitation medicine and physiotherapy, University Hospital Brussels, Brussels, Belgium.

^6^ Société Scientifique de Médecine Générale (SSMG), 1060 Brussels, Belgium.

^7^ Domus Medica, Antwerp, Belgium.

^8^ Department of Physical and Rehabilitation Medicine, Cliniques universitaires Saint-Luc, Brussels, Belgium.

^9^ MOVANT research group, Department of Rehabilitation Sciences and Physiotherapy, Faculty of Health Sciences and Medicine, University of Antwerp, Antwerp, Belgium.

*Correspondence to margot.de.kooning@vub.be

Chronic pain training program

## Objectives of the first workshop

1. Understand acute and chronic pain within a biopsychosocial framework
2. Understand the difference between pain and nociception and acute and chronic pain.
3. Recognize that the purely biomedical model is out-of-date and that the biopsychosocial model of pain should be adopted.
4. Assess patients with (chronic) pain comprehensively
   1. Use questionnaires and interviews to identify patients’ biopsychosocial factors which might influence pain experience according to the PSCEBSM model (pain–somatic factors – cognitive factors – emotional factors – behavioural factors – social factors – motivation).
   2. Assess the patients’ resources, obstacles to improvement, and their “readiness to change”.
5. Integrate contemporary pain science into clinical reasoning in patients with chronic pain
   1. Incorporate patients' biopsychosocial factors when making decisions regarding chronic pain type (e.g. nociceptive, neuropathic and/or nociplastic pain), patients’ evaluation and care request.
   2. Design multimodal treatment programs, either mono- or interdisciplinary, according to the patients’ representations, beliefs, expectations and needs, e.g. stress self-management program, graded activity program, graded exposure, education/reassurance, etc.

**Table A1. Program of the first workshop**

| **Phase and duration (±)** | **Objectives per phase** | **Content; Didactic teaching and learning activities** | **Evaluation of objectives per phase** |
| --- | --- | --- | --- |
| 1st phase, 30 minutes | Introducing trainers, participants, and workshop | *Introduction of teachers and participants*  *Identify the motivation and expectations of participants*  *Discuss the objectives of the training program* | Evaluate through questions and discussions |
| 2nd phase  20 minutes | Align objectives of training with expectations of participants  A brief review of basic concepts | *Discuss the experience of the 1st e-learning module*  *A brief review of basic concepts:*  *- Pain is a necessary protective mechanism*  *- Difference between acute and chronic pain*  *- Pain versus nociception*  *- Pain is always real*  *- Biopsychosocial factors influence pain*  *Inform where possible extra time/attention is needed* | Evaluate through questions and discussions |
| Optional: | Review e-learning content if desired by participants | *Discuss content from the e-learning* |  |
| Break |  |  |  |
| 4th phase  45 minutes | Discuss and practice patient assessment | *Discuss and apply pain classification (nociceptive pain, neurological pain, nociplastic pain) and PSCEBSM-model based on a patient case - in pairs, followed by a group discussion.*  *Discuss different patients’ illness perceptions (identity, cause, timeline, consequences, cure and control), possible relevant questionnaires, and conclusions* | Evaluate through questions and discussions |
| 5th phase  45 minutes | Discuss and practice communication during intake/assessment  Assess "readiness to change". | *Discussion about barriers and facilitators to apply behaviour change techniques in clinical practice and the importance of adequate communication*  *Practice communication to elicit language relating to change in the “patient“:*  *- Experience how not to; convince the patient how he must change*  *- Experience how to; ask open questions (examples):*  *-* *What is the main reason for you to change?*  *- What do you hope this change will bring?*  *- How important is this change to you?*  *- How might you approach it?*  *- Do you think it is feasible for you?*  *Apply basic principles of motivational interviewing*  *- Ask open questions*  *- Use a positive approach*  *- Reflect on patients*  *- Summarise*  *Ambivalence: Change in behaviour is influenced by the advantages and disadvantages of the current situation and the new situation. Discuss both situations.* | Evaluate through questions, discussions and observation |
| Break |  |  |  |
| 6th phase  45 minutes | Introduction to pain science education (PSE)  PSE demonstration | *Discussion about who is currently applying PSE, what elements are essential and what tools/materials you can use*  *Present scientific support for PSE*  *Classical demonstration of pain*  *education by the teachers* | Evaluate through questions, discussions and observation |
| 7th phase  45 minutes | PSE skills training | *Practice PSE (while using metaphors);*  *- Explain acute pain to a patient*  *- Explain chronic pain to a patient*  *- Discuss biopsychosocial factors with a patient*  *- Motivate a patient to change beliefs, lifestyle factors and behaviour*  *Exercise to provide PSE based on the patient case* | Evaluate through questions, discussions and observation |
| 8th phase  30 minutes | Improve barriers and facilitators regarding PSE | *Discuss which barriers and facilitators participants foresee for implementing PSE in their clinical practice, and how to reduce these barriers.* | Evaluate through questions and discussions |
| Optional section  60 minutes | Improve knowledge  and skills where desired by participants | *Options:*  *Provide more information or practice skills regarding the biopsychosocial model, PSE, communication techniques or management of patients with chronic pain* |  |
| Final phase  10 minutes | Check if the expectations and objectives of the workshop and participants were achieved  Provide take-home messages and exercises for implementation | *Inform whether expectations and objectives – which were formulated in the 1^st^ phase - have been met*  *Discuss their actions to implement the learned knowledge and skills into clinical practice*  *Share take-home messages and explain exercises for implementation for the upcoming month to practice.* | Evaluate through questions and discussions |

PSCEBSM-model = pain – somatic factors - cognitive factors - emotional factors - behavioural factors - social factors – motivation; PSE = pain science education

## Exercises for implementation

Exercise 1:

- After providing PSE: Immediately after the explanation, have patients tell in their own words how they would explain it to their friends and family.
- After providing PSE: Let patients explain this in their own words during the next treatment.

Exercise 2:

- Use two questionnaires in the treatment plan. Whenever possible, not only during the assessment but also at the end of the entire treatment or after a certain period of time to measure progress objectively.

Exercise 3:

- Give patients assignments or materials to take home to read further on the education of pain at home. Whenever possible, discuss this during the next treatment session.
  - For example, the patient education booklet, available patient videos, watch retrainpain.org, or possibly own materials you use. Ideally, you should offer the patient choices: does the patient prefer to watch an online video or would they rather have a printed brochure to take home? Some patients choose the combination. This is a low-threshold method of shared decision-making that promotes adherence.
  - See: www.paininmotion.be/patients/information-about-persistent-pain

Go to www.paininmotion.be -> Patients -> Information-about-persistent-pain

Exercise 4:

- Explain to a colleague or team the neurophysiology of pain. Explain and discuss the differences between acute pain and chronic pain in a biopsychosocial model, which factors all have an influence and why.
- Discuss with each other which questionnaires you used and/or which questionnaires can improve assessment or treatment.

Exercise 5:

- Use the ICF model in a patient's report (Function, Activities, Participation, Personal factors, Environmental factors).
- Discuss the report with a colleague using the ICF model for more effective communication.

## Second workshop

1. Integrate contemporary pain science into clinical reasoning in patients with chronic pain
   1. Incorporate patients' biopsychosocial factors when making decisions regarding chronic pain type (e.g. nociceptive, neuropathic and/or nociplastic pain), patients’ evaluation and care request.
   2. Design multimodal treatment programs, either mono- or interdisciplinary, according to the patients’ representations, beliefs, expectations and needs, e.g. stress self-management program, graded activity program, graded exposure, education/reassurance, etc.
2. Provide tailored and patient-centred strategies to subacute and chronic pain patients
   1. Educational strategies:
      1. Understand that pain science education (PSE) is a continuous process;
      2. Use communication skills to favour therapeutic alliance;
      3. Master pain neurophysiology and the biology behind different pain mechanisms to be able to explain pain to patients by means of metaphors and tools.
   2. Use a patient-centred approach to define specific goals that are meaningful to the patient.
   3. Manage obstacles to improve the patient’s motivation to change.
   4. Teach patients pain coping skills aligned with the ideas delivered during PSE.
3. Understand the role of HCPs in an interdisciplinary perspective
   1. Understand other healthcare disciplines' roles in successfully managing chronic pain.
   2. Communicate adequately with other HCPs about the management of chronic pain.

**Table A2. Program of the second workshop**

| **Phase and duration (±)** | **Objectives per phase** | **Content; Didactic teaching and learning activities** | **Evaluation of objectives per phase** |
| --- | --- | --- | --- |
| 1st phase  15 minutes | Introduce trainers, participants, and workshop | *Introduction of teachers and participants*  *Identify the motivation and expectations of participants*  *Discuss the objectives of the training program* | Evaluate through questions and discussions |
| 2^e^ phase  30 minutes | Align the objective of training with the expectations of participants | *Inform who implemented the biopsychosocial model, PSE, patient materials in their clinical practice and who completed the exercises*  *Discuss participants’ experiences and their perceived barriers and facilitators*  *Discuss the second e-learning module*  *Inform where possible extra time/attention is needed* | Evaluate through questions and discussions |
| Optional: Review  topics | *If desired by the participants, extensive discussion about previous content* | *Extensive discussion about content from the e-learning modules and the first workshop* |  |
| 3^e^ phase  30 minutes | Improve patient assessment and develop a treatment plan | *Apply the international classification of functioning, disability and health model (ICF-model) to differentiate between types of pain and assess biopsychosocial factors influencing the health problem of a patient case.*  *Determine which biopsychosocial factors are important factors for the participant to address in his/her treatment plan* | Evaluate through questions, discussions and observation |
| Break |  |  |  |
| 4^e^ phase  60 minutes | Improve PSE and motivational interviewing skills | *Practice PSE with extra attention on explaining:*  *- the influence of stress, beliefs, lifestyle factors, and environmental factors on pain*  *- How influencing factors not only influence pain but also each other*  *- Results from medical imaging*  *Practice in groups of 3 so an observer provides detailed feedback, followed by a class discussion* | Evaluate through questions, discussions and observation |
| 5 ^e^ phase  45 minutes | Improve communication skills including motivational interviewing | *Discuss (stages of) behavioural change*  *Motivational interviewing skills:*  *- Ask for permission*  *- Elicit prior knowledge (What do you already know about X?)*  *- Share information (in chunks)*  *- Elicit meaning (What does X mean to you?)*  *Practice responding to a list of patient statements/quotes* | Evaluate through questions, discussions and observation |
| Break |  |  |  |
| 6^e^ phase  40 minutes | Setting patient-centered goals | *Discuss and write down specific and realistic treatment goals (SMART; specific, measurable, achievable, realistic, time-bound) for the patient case with chronic pain with a focus on:*  *- Activity, participation, self-management and quality of life*  *- Patient-centered*  *- shared-decision making with the patient* | Evaluate through questions, discussions and observation |
| 7^e^ phase  90 minutes | Developing a treatment plan with a mono/multidisciplinary approach  Identification of roles between different disciplines | Participants develop a treatment plan for a patient case.  Discussion about mono-/multidisciplinary treatment plans and the roles of different disciplines  Discuss commonly applied treatment modalities and advice:  - PSE  - (Graded) exposure  - (Graded) activity  - Lifestyle coaching (e.g. sleep, stress, nutrition)  - Hands-on techniques  - Medication  - Medical imaging  - Work absenteeism | Evaluate through questions, discussions and observation |
| 8^e^ phase  20 minutes | Create awareness about communication with healthcare providers | *Inform when participants communicate with other HCPs and what information they ask/share*  *Discuss how this communication can be improved*  *Discuss the integration of ICF into their reports* | Evaluate through questions and discussions |
| Optional phase | *Improve knowledge*  *Practise skills* | *Improve knowledge or practise skills*  *based on the needs of the participants* |  |
| Final phase  30 minutes | Check if the expectations and objectives of the workshop and participants were achieved  Provide key messages | *Inform whether expectations and*  *objectives have been met*  *Discuss their actions to implement the learned knowledge and skills into clinical practice* | Evaluate through questions and discussions |

PSE = pain science education; ICF-model = International Classification of functioning disability and health model; SMART = Specific, Measurable, Achievable, Realistic, Time-bound;

## Key messages at the end of the chronic pain training program

- Acute and chronic pain are different
- A biopsychosocial perspective is essential
- Differentiate between nociceptive, neuropathic and neuroplastic pain
- Psychosocial factors are greater predictors of pain than biomedical factors (but biomedical factors are still important!)
- Apply a patient-centred approach
- Provide pain science education (unless the patient does not want it)
  - Reduce "danger"
  - Pain does not equal harm
- Use motivational interviewing
- Focus on change in lifestyle factors
  - Activate lifestyle
  - Focus on participation
  - Improve the quality of life
- Stimulate self-management
- Monodisciplinary treatment plans can be effective, but multidisciplinary are more beneficial
